# Influence of Adverse Drug Reactions on Treatment Success: Prospective Cohort Analysis of HIV-Infected Individuals Initiating First-Line Antiretroviral Therapy in India

**DOI:** 10.1371/journal.pone.0091028

**Published:** 2014-03-10

**Authors:** Anita Shet, Jimmy Antony, Karthika Arumugam, Sunil Kumar Dodderi, Rashmi Rodrigues, Ayesha DeCosta

**Affiliations:** 1 Department of Pediatrics, St. John’s Medical College Hospital, Bangalore, India; 2 Division of Public Health and Infection, St. John’s Research Institute, Bangalore, India; 3 Division of Biostatistics, St. John’s Research Institute, Bangalore, India; 4 National AIDS Control Organization, Ministry of Health and Family Welfare, Government of India, New Delhi, India; 5 Department of Community Medicine, St. John’s Medical College Hospital, Bangalore, India; 6 Division of Global Health, Karolinska Institutet, Stockholm, Sweden; Temple University School of Medicine, United States of America

## Abstract

**Introduction:**

Adverse drug reactions related to antiretroviral therapy (ART) remain a challenge in resource-limited settings, often causing significant morbidity and impaired adherence leading to treatment failure. This 2-year prospective study aimed to describe patterns and predictors of adverse reactions to first-line ART and assess the impact of these events on treatment success.

**Methods:**

Between 2010–2013, 321 ART-naïve eligible adults were enrolled at two clinics in southern India. ART regimens included zidovudine or stavudine plus lamivudine, plus nevirapine or efavirenz. Pill count adherence, immunological and virological monitoring, and laboratory-based adverse drug reactions were measured prospectively and analyzed.

**Results:**

Among 321 patients in the study, 289 (90.0%) patients experienced at least 1 adverse reaction, and 85 (26.5%) experienced at least 1 severe reaction. The incidence rate was 52 and 15 per 100 person-years for all reactions and severe reactions respectively. The cumulative incidence of zidovudine-induced anemia was 37.1% over 2 years. At 12 and 24 months, the proportion of patients with optimal adherence, undetectable viral load and CD4 counts >350 cells/mm^3^ were similar between patients who experienced or did not experience severe adverse drug reactions.

**Conclusions:**

Our results highlight the high frequency of ART-related adverse drug reactions among individuals initiating first-line ART in India, underscoring the importance of detailed counseling and monitoring for maintaining ART durability. Severe drug-induced anemia needs to be addressed urgently with alternative first-line agents, and close laboratory surveillance. High treatment efficacy despite decreased drug safety seen here may be because patients have limited treatment options. Our results support the use of currently recommended safer first-line ART regimens that minimize the risk of severe life-threatening toxicities and provide for a better quality of life.

**Trial registration:**

ISRTCN Registry: ISRCTN79261738.

## Introduction

The widespread accessibility of antiretroviral therapy has transformed HIV into a chronic manageable disease with prolonged survival times. As with any chronic therapy, drug-related toxicities remain a major challenge in resource-limited settings due to a limited formulary and inadequately trained personnel [1], [2]. Adverse drug reactions (ADRs) can often cause significant morbidity among individuals on antiretroviral therapy (ART), occasionally leading to mortality. Indeed, treatment-limiting drug toxicities can add an extra layer of complexity in the management of HIV by impairing patient adherence to treatment, leading to inferior clinical outcomes and higher cost to the public health system [3], [4].

Despite high disease burden, India has made remarkable strides in HIV control and management, led by National AIDS Control Organisation (NACO). As of December 2012, NACO runs 380 ART centers nationwide that offer systematic HIV care, drugs free of cost, and most importantly, a detailed counseling algorithm for psychosocial support and management of adverse reactions, with a deep emphasis on ART adherence [5]. There are 450,000 people accessing medical care at these centers throughout the nation, and these numbers are expected to increase when the World Health Organization-recommended new CD4 threshold of 500 cells/mm^3^ for initiating ART is adopted [6]. In this context, it becomes critical to have a deep understanding of factors that can contribute towards treatment success. Anchored within this conceptual framework of the public health system in India, we designed this study to enhance our knowledge of the occurrence and impact of ADRs. In this prospective analysis, we aimed to describe incidence, timing, intensity and predictors of ADRs to first-line ART within 2 years of ART initiation, and their impact on treatment success.

## Materials and Methods

### Study Population

**Figure 1:**
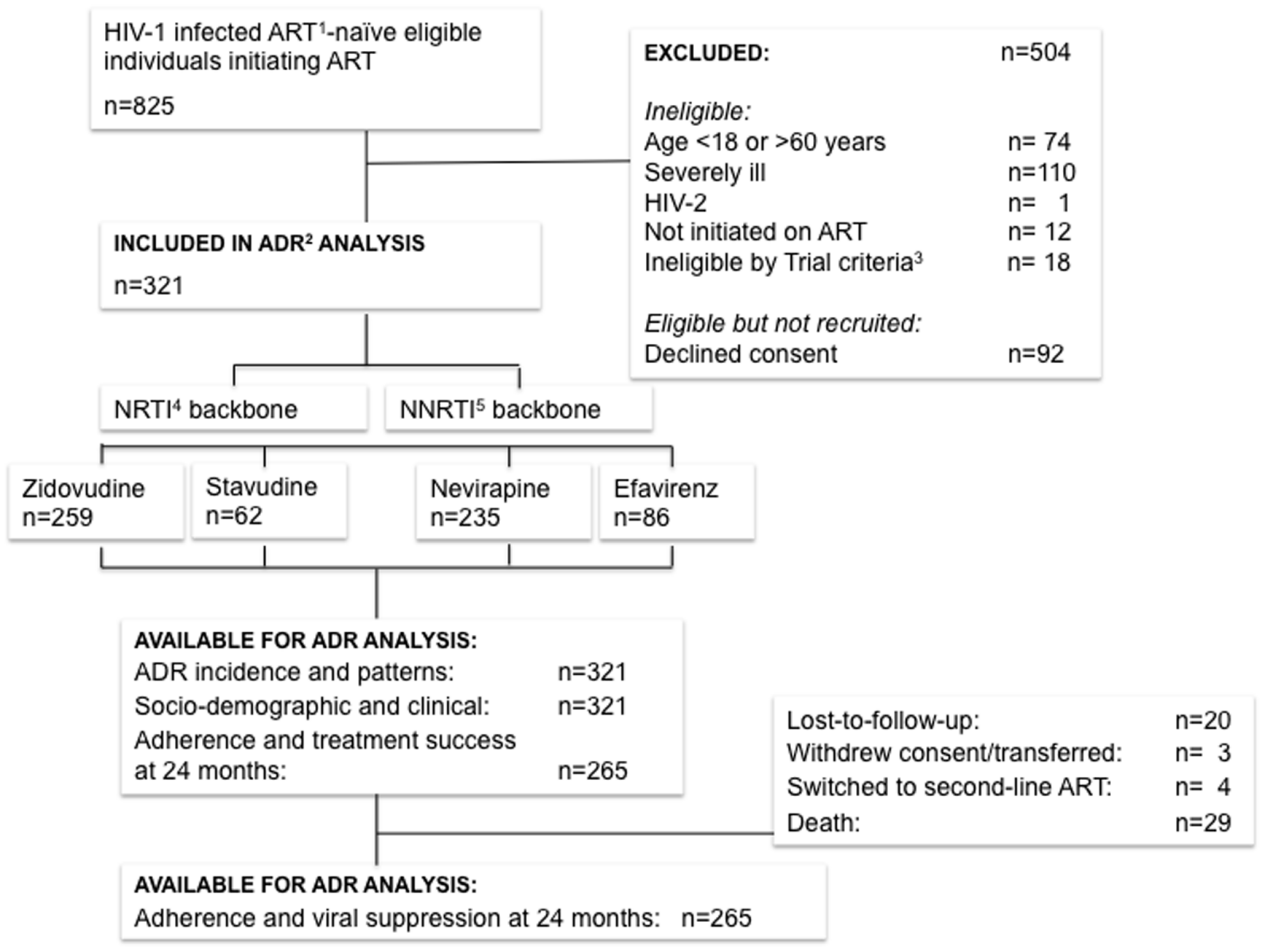
Recruitment flowchart and study outcomes. ^1^ART: Anti- Retroviral Treatment. ^2^ADR: Adverse Drug Reaction. ^3^Trial exclusion criteria: Participant in same household, no available phone network. ^4^NRTI: Nucleoside Reverse Transcription Inhibitor. ^5^NNRTI: Non-Nucleoside Reverse Transcription Inhibitor.

The prospective study of ADRs to antiretroviral treatment was nested within the HIV-India Trial (HIVIND), an open randomized controlled trial, which began in 2010 with the objective of evaluating the efficacy of a mobile phone-based intervention on adherence to ART in the Indian setting [7]. Patients either received the mobile phone-based intervention or not, and the primary outcome was virological failure in both arms. Patients were not randomized based on ART regimens and all patients received the standard ART regimens that were prescribed according to the existing Indian national guidelines [8]. From July 2010 to August 2011, patients at two selected sites in South India, were screened and enrolled in the study after written informed consent. Eligibility criteria included HIV-1 positive individuals aged between 18–60 years who were ART-naïve and willing to start ART as per the Indian national guidelines [7] (Figure 1). Individuals were excluded if they were severely ill (Karnofsky score <70) or were unwilling to come for all study visits. Patients were followed in the trial for a period of 2 years or until they withdrew consent, died, or were lost to follow-up, and the trial concluded in August 2013.

### Study Sites

Two NACO-representative centers in Karnataka, South India were selected for this study. The first, St. John’s Medical College Hospital, Bangalore is a missionary teaching hospital with an ART center that is run as a public-private partnership and manages approximately 1,500 HIV-infected patients each month. The second site, Krishna Rajendra Hospital, Mysore, is a state-owned tertiary-level teaching hospital, with an ART center that manages approximately 4,500 patients each month.

### Ethics Statement

Ethical approvals for the conduct of the trial were obtained from the St. John’s Medical College Hospital Institutional Ethical Review Board, and from the Mysore Medical College and Research Institute Ethical Board. Written informed consent was obtained from all participants prior to enrollment.

### Treatment

At the time of enrollment, ART management was steered by the 2007 NACO guidelines [8]. First-line ART included a nucleoside reverse transcriptase inhibitor (NRTI) backbone consisting of zidovudine (or stavudine if baseline hemoglobin was <10 gm/dl) plus lamivudine; and a non-nucleoside reverse transcriptase inhibitor (NNRTI) drug which included nevirapine (or efavirenz if the patient was on concomitant anti-tuberculous treatment). These medicines were supplied as generic fixed-dose combination pills, and were provided free of cost to patients after intense counseling in sessions spread over 1–3 weeks. During this counseling process and at the follow-up clinic visits, major emphasis was placed on adherence promotion, discussion of barriers to adherence, including possible adverse drug reactions, as well as a wide range of psychosocial issues.

### Clinical Assessments and Laboratory Measurements

The clinical personnel at each of the clinics included a full complement of a medical officer, nurses, counselors, pharmacist, peer worker and several research assistants. After ART initiation, ADR events, if present, were recorded using a specially designed form. ADR events occurring in the interim between 2 consecutive study visits were recorded in the ADR form and indicated as an “unscheduled” study visit. Adherence to ART was measured quantitatively at every study visit using the pill count method. ADRs were monitored both by means of symptoms reported by patients, and active surveillance via clinical and laboratory monitoring of participants in the trial. The medical officer assessed each patient at every study visit. Clinical symptoms and signs, as well as possible adverse events were meticulously probed for and recorded. CD4 counts and viral load assessments were performed as per the HIVIND trial protocol [7]. Hemoglobin measurements, liver enzyme tests and serum biochemical analyses were performed every 6–12 months, and when clinically indicated. All ADRs diagnosed were reviewed independently by a trained study nurse and the lead study investigator in order to minimise bias.

### Definitions

An adverse drug reaction was defined as a response to a drug which is noxious and unintended and which occurs at doses normally used in humans [9]. Symptoms reported by the participants, as well as laboratory abnormalities were defined as ADRs as per their clinical definitions outlined in the Division of Acquired Immunodeficiency Deficiency Syndrome (DAIDS) Table for Grading the Severity of Adult and Pediatric Adverse Events (DAIDS 2004) [10]. The severity of ADRs was graded as follows: mild (grade 1), moderate (grade 2), severe (grade 3) and life-threatening (grade 4) [10].

The likelihood that the adverse event was indeed linked to the antiretroviral agent was estimated by the physician and recorded as “definitely related”, “probably related”, “possibly related” and “not related” based on principles laid down by prior workers [11] and the World Health Organization pharmacovigilance definitions [9]. For analysis, clinical and laboratory abnormalities were defined as ADRs “related” to the antiretroviral agent if the physician estimated the link as either definite or probable. Abnormalities estimated as possible or not related were not considered as an ART-related ADR.

Anemia at baseline was defined as hemoglobin<12 gm/dl in females, and Hb<13 gm/dl in males [12], while anemia as an ART-related adverse event was graded according to the DAIDS criteria (grade 1: Hb 8.5–10 gm/dl; grade 2: Hb 7.5–8.4 gm/dl; grade 3: Hb 6.5–7.4 gm/dl; grade 4: Hb<6.5 gm/dl). Coinfections considered for analysis included tuberculosis, hepatitis B and C virus infection. Concomitant drugs that were considered in the analysis were cotrimoxazole, anti-tuberculosis therapy and fluconazole. Optimal adherence (defined as pill count adherence >95%) and treatment outcomes were measured using CD4 absolute counts and HIV-1 viral load tests.

### Statistical Analysis

Data from the HIVIND database were extracted for analysis and imported to SPSS (v21, IBM). Analysis was by intention-to-treat. System-specific ADRs were described in relation to the causal drug, and the relative risk for selected ADR outcomes was calculated from incidence rates. Logistic regression models were fit to assess the relationship between patient factors and ADR events. Individuals were grouped based on whether they experienced an ADR, and the ADR severity. Adherence and treatment success was compared between patients who experienced, or did not experience severe ADRs using t-test and chi square analysis. All reported p values were two-sided and statistical significance was defined as p<0.05.

## Results

### Baseline Characteristics

Between July 2010 and August 2011, 825 HIV-1 infected ART-naïve individuals initiating ART were screened at the study sites. Patients were excluded if the trial criteria were not fulfilled, and 321 patients were included in the ADR analysis (Figure 1). Of these, 265 were followed for 2 years, and the remaining 56 for shorter periods because of death (29), lost-to-follow-up (20), withdrawn consent (3) and switched to second-line ART (4). The mean age of patients was 37±8 years and 41% were females (Table 1). Among 321 patients included in the study, 81% were on a zidovudine-based regimen, while the most common NNRTI backbone was nevirapine (73%).

**Table 1:** Baseline characteristics of patient population.

Characteristics		n = 321	%
Sex	Male	189	58.9
	Female	132	41.1
Age	18–34 yrs	130	40.5
	35–60 yrs	191	59.5
Site	Bangalore	158	49.2
	Mysore	163	50.8
Baseline anemia			
	Yes	82	25.5
	No	239	74.5
Nutritional status BMI	<18.5	123	38.3
	> = 18.5	198	61.7
WHO Clinical stage	Mild	173	53.9
	Severe	148	46.1
CD4 count (cells/mm^3^)	<250	251	78.2
	> = 250	70	21.8

### Incidence and Pattern of ADRs

Among the 321 patients in the study, 289 (90%) patients experienced at least 1 ADR, and 85 (26.5%) experienced at least 1 severe event. The total duration of follow-up was 558 person-years, yielding an incidence rate of ADR-experiencing individuals as 52 per 100 person-years (Table 2). The corresponding value for severe ADRs was 15 per 100 person-years. It was common for patients to report multiple ADRs; 103 patients had 2–3 ADRs, and 121 patients had >4 ADRs. Women were twice as likely to experience adverse events compared to males (RR 2.27, 95%CI 1.06, 4.84) (Table 3).

**Table 2:** Incidence rate of adverse drug events (ADRs).

	All ADRs		Severe ADRs	
	Total number of events	Incidence per 100 person-years	Total number of events	Incidence per 100 person-years
Overall ADRs	979	176	99	18
Zidovudine-related	508	91.0	53	9.5
Stavudine-related	77	13.8	13	2.3
Lamivudine-related	24	4.3	2	0.4
Nevirapine-related	143	25.6	19	3.4
Efavirenz-related	79	14.2	9	1.6

**Table 3:** Gender-related incidence of adverse drug reactions (ADR).

	Men number of ADR events, (%)	Women number of ADR events, (%)	RR^1^	95% CI^2^
***All ADRs***	482 (95.6%)	497 (98.0%)	2.27*	1.06, 4.84
***Zidovudine- related***	229 (47.5%)	279 (56.1%)	1.41*	1.09, 1.82
***Stavudine-related***	37 (7.7%)	40 (8.0%)	1.05	0.66, 1.68
***Nevirapine-related***	59 (11.9%)	84 (17.4%)	1.56*	1.10, 2.22
***Efavirenz-related***	43 (8.9%)	36 (7.2%)	0.80	0.50, 1.27

1 RR: Relative Risk;

2 CI: Confidence Interval.

*significant risk.

#### Timing of ADR

The median onset of ADR events was 18 weeks (IQR 4, 48). Fifty percent of all ADRs took place within the first 3 months of initiating ART, and 59% took place within the first 6 months. Nevirapine-related ADRs (RR 2.38, 95%CI 1.67, 3.45) and zidovudine-related anemia (RR 2.50, 95%CI, 1.54, 5.0) were more likely to occur in the first 6 months of ART initiation, and stavudine-related ADRs, particularly dyslipidemias were later manifestations that occurred after 6 months of ART (RR 0.22, 95%CI 0.12, 0.44).

#### Relationship of adverse events with antiretroviral agents

Zidovudine was responsible for the majority of adverse events; 58.7% of all ADRs were linked to zidovudine. Stavudine, nevirapine and efavirenz contributed 9.6%, 19.6% and 12.1% of ADRs, respectively. Women were more susceptible to zidovudine-related ADRs compared to males, even after adjusting for baseline anemia and low body mass index (RR 1.46, 95%CI 1.13, 1.89).

#### Distribution of ADRs

The most common clinical ADRs found within this cohort were gastrointestinal disturbances (15.8%) (Table 4). These were short-term effects that resolved within a median period of 8 weeks. Other commonly reported ADRs were generalized fatigue (10.7%), rash (6.3%) and central nervous system disturbances such as headache, dizziness and insomnia (8.4%). Laboratory abnormalities attributed to ART included elevated liver enzymes (17.1%), anemia (10.3%), neutropenia (8.2%) and dyslipidemia (8.4%). Among severe ADRs, the most prominent was anemia (30.3%), followed by neutropenia (17.2%), hepatic enzyme elevation (13.1%) and rash (10.1%).

**Table 4:** Distribution and patterns of adverse drug reactions (ADR).

			n (%)			
Type of ADRs	All ADRs (n = 979)	Severe ADRs (n = 99)	Zidovudine-related ADRs (n = 508)	Stavudine-related ADRs (n = 77)	Nevirapine-related ADRs (n = 143)	Efavirenz-related ADRs (n = 79)
Gastrointestinal: nausea,vomiting, abdominal pain	155 (15.8)	2 (2.0)	137 (27.0)	11 (14.3)	23 (16.1)	6 (7.6)
Anemia	101 (10.3)	30 (30.3)	100 (19.7)	1 (1.3)	_	_
Neutropenia	80 (8.2)	17 (17.2)	80 (15.7)	_	1 (0.7)	_
Thrombocytopenia	21 (2.1)	4 (4.0)	20 (3.9)	1 (1.3)	_	_
Hepatic: elevated liver enzymes± hyperbilirubinemia	167 (17.1)	13 (13.1)	_		115 (80.4)	52 (65.8)
Dermatological: rash	62 (6.3)	10 (10.1)	2 (0.4)	1 (1.3)	43 (30.1)	19 (24.1)
Dermatological: skin & nail hyperpigmentation	86 (8.8)	_	86 (16.9)	_	_	_
Central nervous system:headache, dizziness, insomnia	82 (8.4)	5 (5.1)	41 (8.1)	2 (2.6)	2 (1.4)	28 (35.4)
Peripheral neuropathy	23 (2.3)	4 (4.0)	_	23 (29.9)	_	_
Metabolic: dyslipidemia	82 (8.4)	9 (9.1)	36 (7.1)	39 (50.6)	1 (0.7)	13 (16.5)
Metabolic: lactatemia	14 (1.4)	2 (2.0)	_	13 (16.9)	_	_
Pancreatitis	1 (0.1)	1 (1.0)	_	1 (1.3)	_	_
Generalized: Fatigue,malaise, myalgia	105 (10.7)	2 (2.0)	91 (17.9)	5 (6.5)	13 (9.1)	4 (5.1)

#### Outcome of ADRs

Among the 979 ADR events that were recorded, 70% resolved completely within a median period of 4 weeks. Of the remaining ADRs that were classified as ongoing at the time of study conclusion, almost 80% consisted of dyslipidemia, skin and nail hyperpigmentation attributable to zidovudine, and generalized fatigue. There were 2 deaths directly related to ADRs; one was secondary to severe lactic acidosis occurring within 3 months of ART initiation and attributable to stavudine. The second death was due to severe anemia developing within 3 weeks of initiating zidovudine. The ART regimen was continued without interruption in 81% of the patients experiencing ADRs. Among 3%, ART was temporarily withheld until the ADR was resolved. In the remaining 16% of patients, the ADR event necessitated treatment modification to a different regimen.

#### Anemia

There were 101 events of drug-related anemia, yielding a cumulative incidence of 37.1% over 2 years (96 newly anemic patients among 259 who were on zidovudine). Among these, a third experienced severe anemia defined as Hb<7.5 (DAIDS grade 3 & 4). Patients on zidovudine had 22 times higher risk of developing anemia compared to those on other regimens (RR 21.7, 95%CI 8.75, 53.89). Concomitant use of cotrimoxazole increased risk of development of anemia (RR 1.75, 95%CI 1.15, 2.70). Logistic regression analysis showed that baseline anemia, low BMI and female sex did not have any effect on the development of anemia, underscoring the theory that zidovudine-induced anemia was an idiosyncratic reaction. Median week of onset of severe anemia was 12 weeks (IQR 2, 22). Fifteen patients were hospitalized for anemia management and 7 received blood transfusion. The death of one patient was directly attributable to severe anemia.

#### Predictors of ADRs

Patients with baseline CD4 counts greater than 250 cells/mm^3^ were twice as likely to develop a severe ADR compared to those with no or mild ADRs (adjusted RR 2.49, 95%CI 1.34, 4.61)(Table 5). Compared to stavudine, patients on zidovudine had a higher risk of experiencing a severe ADR (RR 1.74, 95%CI 1.22, 3.69). Age, gender, baseline clinical features, co-infections and concomitant medications were not significantly associated with developing a severe ADR. Similar results were obtained when the comparison groups included patients with no ADRs and those with at least 1 ADR without considering severity.

**Table 5:** Multivariate regression analysis of predictors of adverse drug reactions (ADRs).

		No ADR or Mild ADR	Severe ADR	Adjusted RR	95% C.I.	
*Socio-demographic features*						
**Sex**	Male	145	44			
	Female	91	41	1.35	0.74	2.4
**Age (years)**	<35	99	31			
	≥35	137	54	1.80	0.99	3.234
**Residence**	Urban	147	55			
	Rural	89	30	0.72	0.41	1.29
**Annual income (USD)** 1	<1000	158	55			
	≥1000	78	30	1.01	0.57	1.81
*General clinical features*						
**Body mass index**	<18.5	92	31			
	≥18.5	144	54	1.02	0.58	1.80
**Baseline anemia**	No	175	64			
	Yes	61	21	1.72	0.93	3.18
**Hepatitis B or C co-infection**	No	227	83			
	Yes	9	2	0.50	0.10	2.51
*HIV-related clinical features*						
**Clinical Stage**	1, 2	123	50			
	3, 4	113	35	0.63	0.33	1.23
**CD4 count (cells/mm** 3 **)**	<250	193	58			
	≥250	43	27	2.49*	1.34	4.61
*HIV-related clinical features*						
**Regimen: NRTI** 2	Stavudine -based	50	12			
	Zidovudine -based	186	73	1.74*	1.22	3.69
**Regimen: NNRTI** 3	Nevirapine-based	175	60			
	Efavirenz-based	61	25	1.16	0.59	2.28
*Concomitant drugs*						
**Anti-tuberculosis therapy**	No	175	64			
	Yes	61	21	1.20	0.54	2.69
**Cotrimoxazole**	No	132	44			
	Yes	104	41	1.20	0.71	2.04

*significant difference seen.

1 USD, United States dollars,

2 NRTI: Nucleoside reverse transcriptase inhibitor,

3 NNRTI: Non- nucleoside reverse transcriptase inhibitor.

#### ADRs and treatment outcomes

The impact of severe ADRs and treatment success was assessed using the parameters of adherence, absolute CD4 count increase, and HIV-1 viral load. Six months after starting ART, mean adherence among those who experienced a severe ADR was significantly lower compared to those who either experienced no ADR or a mild ADR. (93.9% and 97.6%, respectively, p = 0.01). The proportion of optimally adherent (>95%) patients between the two groups at different time points during the follow-up period is shown in Table 6. Beyond the first 6 months, there was no significant difference in the adherence proportions between the two groups of patients. The overall failure rate in the cohort was 11.8% in the first year, and 7.3% in the second year. There was no significant difference in virological failure at 6, 12 and 24 months after ART initiation between patients with and without severe ADRs, indicating that a comparable degree of treatment success was observed irrespective of the presence or severity of ADR. Analysis of the immunological response at the different time points showed no significant differences in absolute CD4 counts in these patient groups (Table 6).

**Table 6:** Treatment response and adverse drug reaction.

	No or mild ADR n, (%)	Severe ADR n, (%)	p value
*Adherence to antiretroviral therapy*			
**Optimally adherent** 1 **patients at 6 months**	187 (85.5%)	59 (73.8%)	0.02*
**Optimally adherent patients at 12 months**	180 (90.9%)	69 (93.3%)	0.61
**Optimally adherent patients at 24 months**	173 (91.5%)	62 (85.7%)	0.16
*Virological response*			
**Viral failure** 2 **at 6 months**	25 (10.1%)	10 (11.5%)	0.94
**Viral failure at 12 months**	22 (12.1%)	15 (11.1%)	0.12
**Viral failure at 24 months**	35 (12.3%)	17 (10.5%)	0.33
*Immunological response*			
**CD4>350 at 6 months**	73 (39.1%)	37 (45%)	0.06
**CD4>350 at 12 months**	82 (49.6%)	34 (44.7%)	0.39
**CD4>350 at 24 months**	99 (61.3%)	41 (68.3%)	0.23

*significant difference seen.

1 Optimally adherent: adherence >95%.

2 Viral failure: viral load >400 copies/ml.

## Discussion

Our study provides evidence of a high degree of adverse drug reactions occurring among Indian patients initiating first-line ART with zidovudine, stavudine or nevirapine-based regimens. Despite a lowered safety profile of these regimens, treatment efficacy remained comparable among those with and without severe ADRs. The finding that treatment success can be independent of the occurrence of drug toxicities has been previously reported [13]. Results from a large multi-centric international trial (PEARLS ACTG Study) involving over 1500 participants in 8 resource-limited countries indicated that although efficacy was equivalent between the tenofovir-emtricitabine-efavirenz and zidovudine-lamivudine-efavirenz regimens, the tenofovir-based regimen had a clear safety advantage, particularly among women [13]. Although acceptable efficacy despite drug toxicity may be seen among patients with limited options, this finding does not preclude the need for close monitoring for adverse events or the use of safer alternative regimens.

Our study revealed a considerably high rate of ART-related ADRs. Ninety percent of the participants experienced at least 1 ADR event, and almost a quarter of these were severe events (DAIDS grade 3 or 4). A similar high incidence of ADRs has been reported only by a few studies; an Iranian study with a 6-month follow-up period reported an ADR incidence of 88% [14]. Surveillance among over 1,000 patients on ART in Nigeria revealed an ADR incidence of 63% among those on zidovudine-based ART [15]. Several studies from the African subcontinent and Europe focusing on ADR-related treatment modification found ADR incidence rates to range from 11–53% [16]–[21]. Indian retrospective studies where similar regimens were used have noted ADR incidence rates of 34–53% [22]–[25]. The high rate of ADRs that we found in our study may be partially explained by the prospective systematic collection of this data within a trial setting and the use of a structured questionnaire.

Our results indicated that female patients had a higher risk of developing ADRs. Several other observational studies have reported a higher risk of drug toxicity developing in women [4], [16], [18], [26] particularly when considering zidovudine-based and nevirapine-based regimens [27]–[29], and may be attributable to gender-related disparity in antiretroviral pharmacokinetics [30]. The relationship between CD4 counts and ADRs has been inconsistent, while some have reported an association of ADRs and higher CD4 counts [29], others have shown lower CD4 counts to be better predictors of ADRs [31] while others have shown equivocal results [19]. These conflicting results may be partially explained by the differing types of ADRs, ART regimens and CD4 count thresholds considered in these studies.

The high incidence of anemia in our study was striking and somewhat disconcerting. The onset of anemia among a third of all those who were initiated on zidovudine-based ART warrants a rethinking of our strategies to combat this often silent but deadly ailment. Other studies have reported lower incidence of anemia even when zidovudine was the predominant NRTI used in the ART regimen. Anemia incidence in a large study in a public health setting in Nigeria was 4% [19], and other studies conducted in Camaroon, Cote d’Ivoire and Uganda reported severe anemia incidence of 3.8–6.6% [17], [32], [33]. In Cambodia, among a cohort of 1180 treatment-experienced patients newly switched to zidovudine, new-onset anemia was 12% [34]. A prospective cohort study in Iran reported drug-related anemia incidence as 22%, and 94% of these patients were on zidovudine-containing regimens [14]. A large pooled study from African, Asian and South American patients showed a high prevalence of anemia development after ART initiation of up to 40%, however the exact distinction between nutritional anemia and drug-induced anemia was not clear [35]. While previous reports from India have reflected a low incidence of drug-related anemia (5.4%) [22], a more recent prospective study from Delhi found that the incidence of zidovudine-induced anemia was 34.5% [36]. No specific baseline variable was found to be strongly associated with anemia in our study, however an important observation was the high rate of hospitalization and the occurrence of one preventable death among those with severe anemia. With the 2013 WHO guidelines relegating zidovudine to second- or alternative-line ART, we can expect to see less of these serious adverse effects [37]. Our results make a compelling case for short-term intense monitoring of hemoglobin and appropriate management whenever initiating zidovudine-containing ART.

Drug–related toxicities are associated with poor adherence and subsequent treatment failure [3], [38], [39]. Inadequate knowledge on the patient’s part and insufficient counseling by the healthcare giver has been cited as reasons for adverse drug reactions contributing towards poor adherence to care [40]. Despite the drop in adherence in the first 6 months in our study, a key observation here is the equalization in adherence measurements in patients grouped by the occurrence and severity of ADRs after the first 6 months. In addition, virologic and immunologic outcomes at 12 and 24 months were not different in individuals with severe ADRs, suggesting that good adherence and successful treatment outcomes can be achieved with adequate counseling and clinical management of drug toxicity manifestations. Investigators from the Swiss HIV Cohort Study observed a moderately high rate of treatment switches or discontinuations due to drug toxicity, but drew similar conclusions that given adequate implementation treatment algorithms, the high frequency of treatment interruptions did not diminish treatment success [41]. Other cohort studies from the UK and US also underline the importance of close monitoring for sustained durability of ART [42], [43].

We acknowledge some limitations in our study. The drug regimens analyzed in this study included the standard regimens prescribed and dispensed to the patients under the national program prior to release of the new 2013 WHO ART guidelines. Misclassification of the cause of the ADRs was a possibility, as our analysis was limited by the relationship that was assigned by the physician assessing the patient during the event. We minimized variations in causality assignment by the use of standard guidelines and by independent assessment by a trained study nurse. It may be argued that the trial setting within which the observational study was conducted may have contributed towards the positive treatment outcomes reported even among patients with severe ADRs. It is important to note here that all counseling was done by the regular clinic staff, while research staffs were only involved in data collection and conducting the trial.

Despite these limitations, our study has certain notable strengths. First, the analysis of ADRs was based on active surveillance of clinical and laboratory parameters, and delivered detailed and accurate information on ART-related drug toxicity. Second, the prospective longitudinal nature of the study provided minimal loss of data. Third, few studies on ADRs have combined data on adherence, immunological and virological measurements to study the impact of drug toxicity, and this is a major advantage with this study. Finally and most importantly, the study was based within the Indian National ART program setting and has produced information that can be generalizable and has public health relevance nationally and to some extent, globally.

## Conclusion

Although high incidence of adverse drug reactions remains a reality in ART administration for HIV care, detailed counseling during the early stage of ART initiation, along with good clinical and laboratory monitoring, can go a long way in maintaining the durability of first-line regimens in resource-limited settings. The high incidence of drug-induced anemia in this population should be urgently addressed with alternative first-line agents, or close laboratory monitoring. High treatment efficacy despite decreased drug safety may be seen among patients with limited options; nevertheless patients deserve superior ART regimens that curtail the risk of severe life-threatening toxicities. National programs may do well to adopt the newer, safer alternative ART agents proposed by the 2013 WHO guidelines as first-line options in lieu of zidovudine and nevirapine [37]. Future operational research can incorporate long-term pharmacovigilance and assessment of the robustness of alternate first and second-line therapy.

## Supporting Information

File S1 **HIVIND Trial Protocol.**
(PDF)
